# The role of circulating microRNAs for the diagnosis of hepatitis B virus-associated hepatocellular carcinoma with low alpha-fetoprotein level: a systematic review and meta-analysis

**DOI:** 10.1186/s12876-020-01345-5

**Published:** 2020-07-31

**Authors:** Cheng Peng, Zhuonan Li, Zishan Xie, Zhanpeng Wang, Yanshuo Ye, Bo Li, Wei Li

**Affiliations:** 1 Department of Hepatobiliary-Pancreatic Surgery, China-Japan Union Hospital of Jilin University, 126 Xiantai Street, Changchun, 130033 China; 2 Department of Hepatobiliary-Pancreatic Surgery, Third Xiangya Hospital of Central South University, Changsha, 410013 China; 3 Department of Plastic Surgery, China-Japan Union Hospital of Jilin University, Changchun, 130033 China; 4 Department of Ultrasonography, the Eighth Affiliated Hospital of Sun Yat-sen University, Shenzhen, 518020 China; 5 Department of Epidemiology, School of Public Health of Jilin University, Changchun, 130021 China

**Keywords:** miRNAs, HCC, Low AFP, Diagnosis, Meta-analysis

## Abstract

**Background:**

Alpha-fetoprotein (AFP) has been widely used for many years as a serum marker for hepatocellular carcinoma (HCC). However, AFP has been recognized as having poor sensitivity. More and more studies have concluded that circulating microRNAs (miRNAs) might be a promising biomarker that could complement AFP. However, the diagnostic ability of circulating miRNAs has varied among the studies. Therefore, we performed the present meta-analysis to appraise the diagnostic performance of circulating miRNAs as a biomarker for hepatitis B virus-associated HCC (HBV-HCC) patients with low AFP levels.

**Methods:**

We performed a systematic review and meta-analysis of the published literature to assess the diagnostic accuracy of circulating miRNAs in differentiating HBV-HCC patients with low AFP levels from non-HCC controls.

**Results:**

Circulating miRNAs showed promising potential in the diagnosis of HBV-HCC patients with low AFP levels. In the low-AFP HBV-HCC patients, the area under the curve (AUC) was 0.88 (95% confidence interval [CI]: 0.84–0.90). The pooled sensitivity and specificity were 0.84 (95% CI: 0.78–0.88) and 0.76 (95% CI: 0.69–0.83), respectively.

**Conclusions:**

The detection of circulating miRNAs provides a valuable method for the diagnosis of HBV-HCC in patients with low AFP levels.

## Background

Hepatocellular carcinoma (HCC) comprises 75 to 85% of cases of primary liver cancer and ranks as the sixth most commonly diagnosed cancer and the fourth most common cause of cancer-related deaths worldwide [1]. Hepatitis B virus-associated HCC (HBV-HCC) accounts for more than 80% of HCC cases in China and at least 50% of HCC cases worldwide [2].

HCC patients are often diagnosed at the late stage due to lack of specific symptoms, resulting in a relatively low 5-year survival rate of less than 30% worldwide [3], but it can be increased to 60 to 70% for early-stage HCC patients who receive surgical intervention [4]. Therefore, populations at high risk for HCC are recommended to undergo surveillance by abdominal ultrasound (US) plus alpha-fetoprotein (AFP) level screening for HCC, which leads to a better prognosis [5]. However, a large-scale, multicenter study in China showed that the sensitivity of AFP was only 68% in identifying HCC [6], which is not very satisfactory. In such a clinical setting, liquid biopsy has emerged as a promising strategy for the diagnosis of HCC [7], especially for patients with low AFP levels (AFP < 400 ng/ml) or even patients who are AFP-negative (AFP < 20 ng/ml).

The detection of circulating microRNAs (miRNAs) is a part of liquid biopsy. MiRNAs are a group of non-coding endogenous RNAs, which form complex post-transcriptional networks and regulate the process of liver cirrhosis [8], carcinogenesis of HCC [9], and drug resistance [10]. Circulating miRNAs can sustain stability and avoid being degraded thanks to their various existing forms in the blood, where ribonuclease is richly contained [11]. This indicates that circulating miRNAs are a promising novel HCC diagnostic marker.

In recent years, numerous studies have concluded that the quantitative detection of aberrantly expressed circulating miRNAs may be a novel strategy for the diagnosis of HBV-HCC patients with low AFP level. However, the results varied among studies. Therefore, we conducted the present meta-analysis to summarize the diagnostic performance of circulating miRNAs.

## Methods

### Search strategy and study selection

The process of literature search and study selection was in strict accordance with the PRISMA guideline [12]. We formulated a scientific and complete search strategy to identify studies evaluating the diagnostic efficiency of circulating miRNAs for HBV-HCC patients with low AFP level. Language and publication year were not restricted. The online databases included PubMed, Embase, Cochrane Library, Chinese National Knowledge Infrastructure (CNKI), WanFang Datebase, and VIP. Potential relevant studies were obtained by manual searching based on reference lists of some related reviews. The search terms and search strategy we applied are listed as follows:

- #1: MeSH terms: carcinoma hepatocellular; Entry terms: carcinoma hepatocellular; hepatocellular carcinoma; hepatocellular cancer; hepatocellular tumor; hepatocellular neoplasm; liver cell carcinoma; liver cell cancer; liver cell tumor; liver cell neoplasm; HCC
- #2: MeSH terms: microRNAs; Entry terms: microRNAs; microRNA; miRNA; miRNAs; miR; panel
- #3: MeSH terms: serum; plasma; blood; Entry terms: serum; plasma; blood; circulating; circulatory
- #4: MeSH terms: diagnosis; biomarkers; Entry terms: diagnosis; diagnostic; screen; monitor; detect; predict; predictor; prediction; specificity; sensitivity; marker; biomarkers; AUC; ROC; clinical implication
- #5: #1 AND #2 AND #3 AND #4

A substantial number of records were obtained by online database searching and manual searching. First of all, we conducted a removal of duplicate publications using Endnote X9 software (Clarivate Analytics, Philadelphia, PA, USA). A study was included in the process of title and abstract assessment if it met all the inclusion criteria that we pre-specified: (1) The study population consisted of HBV-HCC patients and non-HCC controls; (2) Diagnostic research was conducted assessing the diagnostic performance of circulating miRNAs as a biomarker for HBV-HCC patients with low AFP levels; (3) The specimen was restricted to plasma, serum or whole blood. Any study without sufficient information or data was excluded from the process of full-text assessment.

### Data extraction

One investigator extracted the related data and inserted the data into a standardized table, while another investigator checked and corrected the data. We extracted the following essential data from the included studies: (1) The name of the leading author, year of publication, region, specimen type, the miRNAs involved in the studies, and their corresponding normalization control; (2) The number of HBV-HCC patients and non-HCC controls as well as their status of basic liver diseases, such as viral hepatitis, cirrhosis and so on; (3) Direct or indirect data which was indispensable for meta-analysis, including the sensitivity (SEN) and specificity (SPE) of studied circulating miRNAs for HBV-HCC, the number of true positive (TP), true negative (TN), false positive (FP), and false negative (FN) results in diagnostic tests, and the information needed for quality assessment.

### Study quality assessment

We applied both the Quality Assessment of Diagnostic Accuracy Studies (QUADAS) [13] and QUADAS-2 [14] tools to conduct the quality assessment of the included studies. QUADAS and QUADAS-2 were introduced in 2003 and 2011, respectively. QUADAS is simple and quick to complete, consisting of a set of 14 questions, each of which should be answered as yes (+ 1), no (− 1), or unclear (0). The corresponding total score is calculated after finishing all the items. It is generally considered that a total score of greater than or equal to 9 indicates a relatively high quality. The QUADAS-2 tool was developed from the widely used QUADAS tool. It evaluates the risk of bias (high, low, or unclear) and concerns about applicability (high, low, or unclear) in four domains including “patient selection”, “index test”, “reference standard,” and “flow and timing”. Any disagreements about the quality assessment were settled through discussion or by consulting an expert. The process of quality assessment and the output of the corresponding chart were finished by the RevMan 5.3 software package (Cochrane Community, London, UK).

### Data synthesis and analysis

The statistical analysis was performed through STATA 14.0 (SataCorp, College Station, TX, USA) and Meta-DiSc 1.4 software [15], including pooled SEN and SPE with 95% confidence interval (CI). We also plotted the summary receiver operating characteristic curve (sROC) to obtain the area under the curve (AUC), which can comprehensively reflect the diagnostic performance of a diagnostic marker.

The heterogeneity of the enrolled studies was estimated using Cochran’s Q test and the value of I^2^. A value of I^2^ less than 50% suggested that the heterogeneity was not significant; we then used the fixed effect model to perform the pooled analysis. A value of I^2^ greater than 50% suggested that the heterogeneity was significant; then the random effects model was applied [15, 16].

In order to identify the possible source of heterogeneity, we first examined the existence of a threshold effect, then conducted sensitivity analysis and subgroup analysis based on some common heterogeneity sources including study design, type of specimen, study design, miRNAs profiling, QUADAS score and type of conference test. Publication bias was assessed by Deeks’ funnel plots [17]. A *p* < 0.05 was considered statistically significant.

## Results

### Characteristics of the included studies

**Fig. 1:**
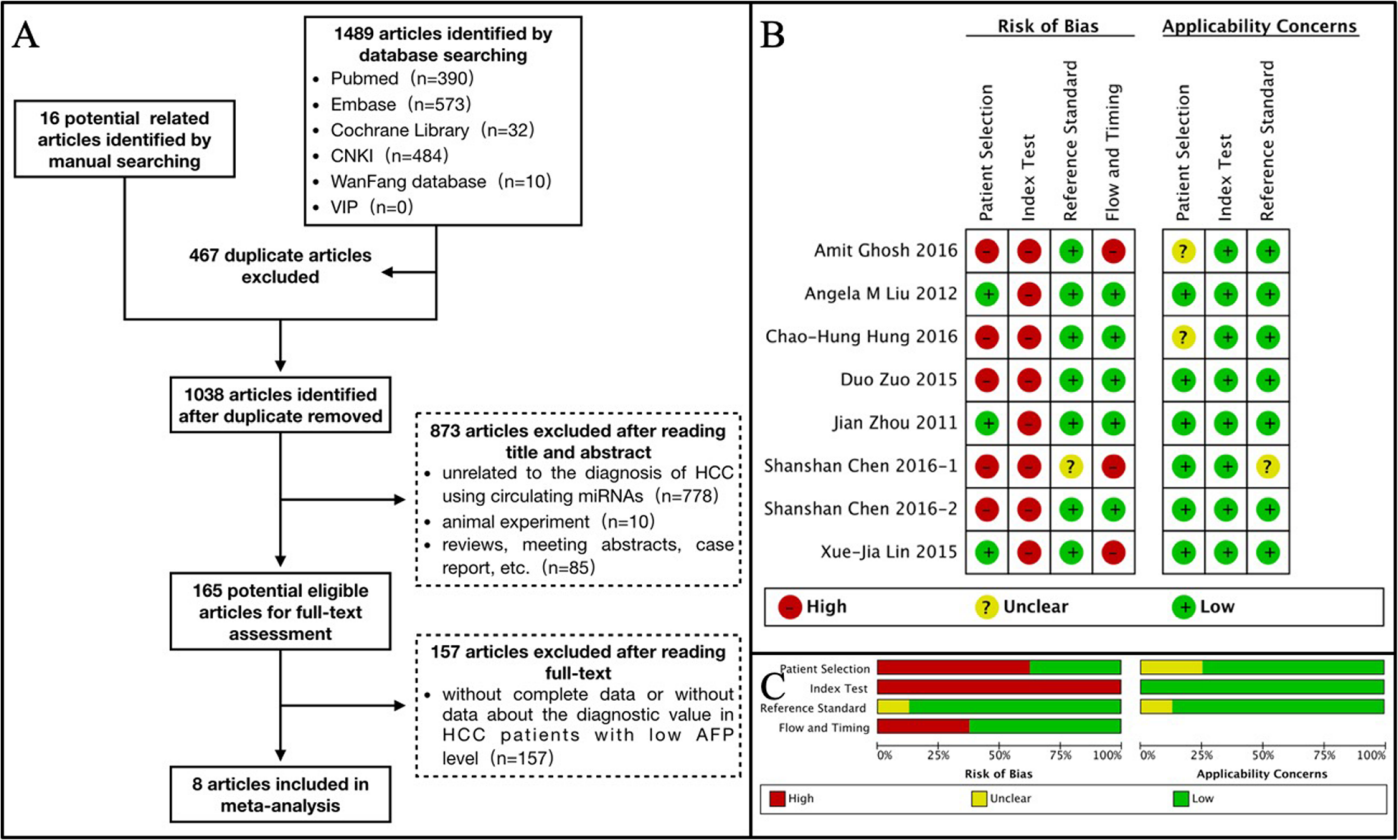
References search strategy and their quality assessment. **a** Flow diagram of study identification and selection for meta-analysis; **b** Risk of bias and applicability concerns summary: review authors’ judgments about each domain for each included study; **c** Risk of bias and applicability concerns graph: review authors’ judgments about each domain presented as percentages across included studies

The literature search yielded a total of 1505 studies by database searching (*n* = 1489) and manual searching (*n* = 16); among these, 467 studies were duplicates and were excluded. We excluded 873 additional studies after reading the titles and abstracts. Hence, only 165 studies were used for full-text assessment; 8 out of the 165 studies were eligible and were ultimately included [18–25]. These included 869 HBV-HCC patients and 1338 non-HCC controls. The flowchart of study identification and selection is shown in Fig. 1a.

Among the 8 included studies, the publication years were 2011 (*n* = 1), 2012 (*n* = 1), 2015 (*n* = 2), and 2016 (*n* = 4). The regions included China (*n* = 7) and India (*n* = 1). The types of study design included case control (*n* = 5) and cohort (*n* = 3). All studies used real-time PCR (RT-PCR) to quantify miRNAs, and the specimens included plasma (*n* = 4) and serum (*n* = 4), which were collected before any treatment. Diagnosis of most of the cases of HCC were established through pathological examination of resected surgical specimens or diagnostic biopsy; other cases were confirmed by imaging examinations. The detailed characteristics of the included studies are listed in Table 1.

**Table 1:** Characteristics of studies included in the meta-analysis

author	year	region	specimen	conference test	index test	study design	markers studied	normalization controls	Low AFP HCC patients		non-HCC controls		QUADAS score	reference
									No.	population	No.	population		
Jian Zhou	2011	China	plasma	histology	RT-PCR	cohort	122, 223, 26a, 27a, 192, 21, 801	miR-1228	139	HBV-HCC (139)	187	HC (66) CHB (68)HBV-LC (53)	10	18
Angela M Liu	2012	China	serum	histology	RT-PCR	cohort	15b, 130b, 21, 183	n.a.	30	HBV-HCC (30)	59	HC (30) CHB (29)	11	19
Duo Zuo	2015	China	serum	histology	RT-PCR	case-control	125b, 223, 27a, 26a	U6	38	HBV-HCC (38)	48	HC (27) CHB (21)	11	20
Xuejia Lin	2015	China	serum	histology or imaging	RT-PCR	cohort	29a, 29c, 133a, 143, 145, 192, 505	cel-miR-67	119	HBV-HCC (119)	438	HC (159) CHB (119) HBV-LC (118) HBV (42)	8	21
Chao-Hung Hung	2016	China	serum	histology	RT-PCR	case-control	122, 7b	U6	92	HBV-HCC (92)	30	HBV-DN (30)	8	22
Amit Ghosh	2016	India	plasma	histology or imaging	RT-PCR	case-control	126, 142-3p	cel-miR-39	18	HBV-HCC (18)	38	HBV-LC (20) CHB (18)	6	23
Shanshan Chen	2016	China	plasma	histology or imaging	RT-PCR	case-control	125b	U6	31	HBV-HCC (31)	158	HC (56) CHB (50) HBV-LC (52)	8	24
Shanshan Chen	2016	China	plasma	histology	RT-PCR	case-control	205	U6	32	HBV-HCC (32)	49	HBV-LC (49)	8	25

*CHB* chronic hepatitis B, HC healthy control, *LC* liver cirrhosis, *n.a*. not available, *HBV* hepatitis B virus infection, *HCC* hepatocellular carcinoma, *HBV-HCC* hepatitis B virus-associated HCC, *DN* dysplastic nodule

The QUADAS scores are listed in Table 1. Three of the studies had a QUADAS score ≥ 9, while 5 of the studies had a QUADAS score < 9. The results of the QUADAS-2 tool are summarized in Fig. 1b and Fig. 1c. In the “patient selection” domain, 5 out of the 9 included studies had not avoided case-control design, which led to a high risk of bias in this section. In the domain of “index test”, the cut-off values of all the included studies were determined by plotting ROC curves with the principle of maximizing SEN and SPE, which resulted in a high risk of bias in this part.

### Summary

**Fig. 2:**
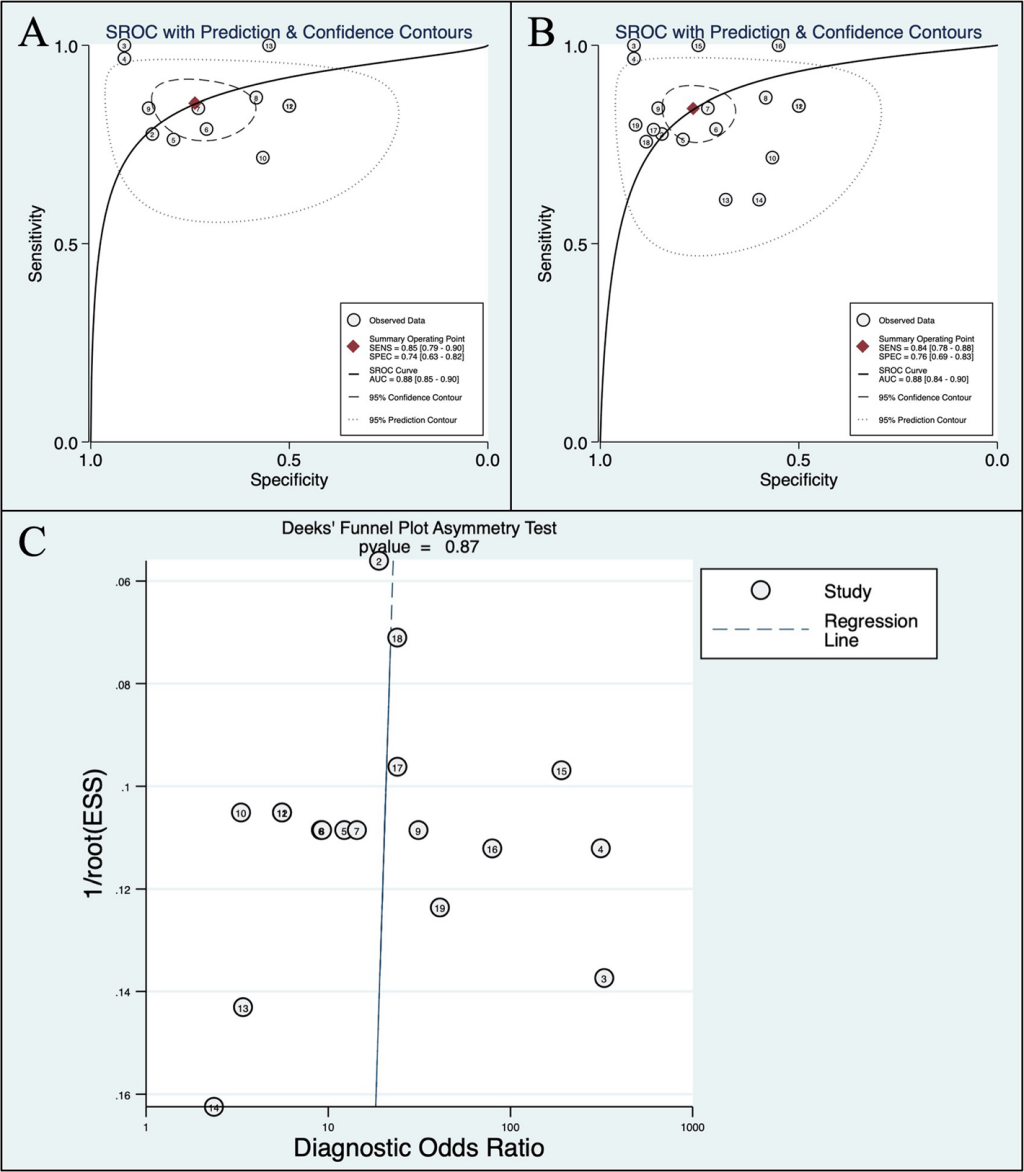
Summary receiver operating characteristic (sROC) curve describing the diagnostic performance of circulating miRNAs. The Deeks’ test detects publication bias of included references. **a** sROC of circulating miRNAs for the diagnosis of HBV-HCC patients with AFP<20 ng/ml; **b** sROC of circulating miRNAs for the diagnosis of HBV-HCC patients with AFP<400 ng/ml; **c** Deeks’ funnel plot

**Fig. 3:**
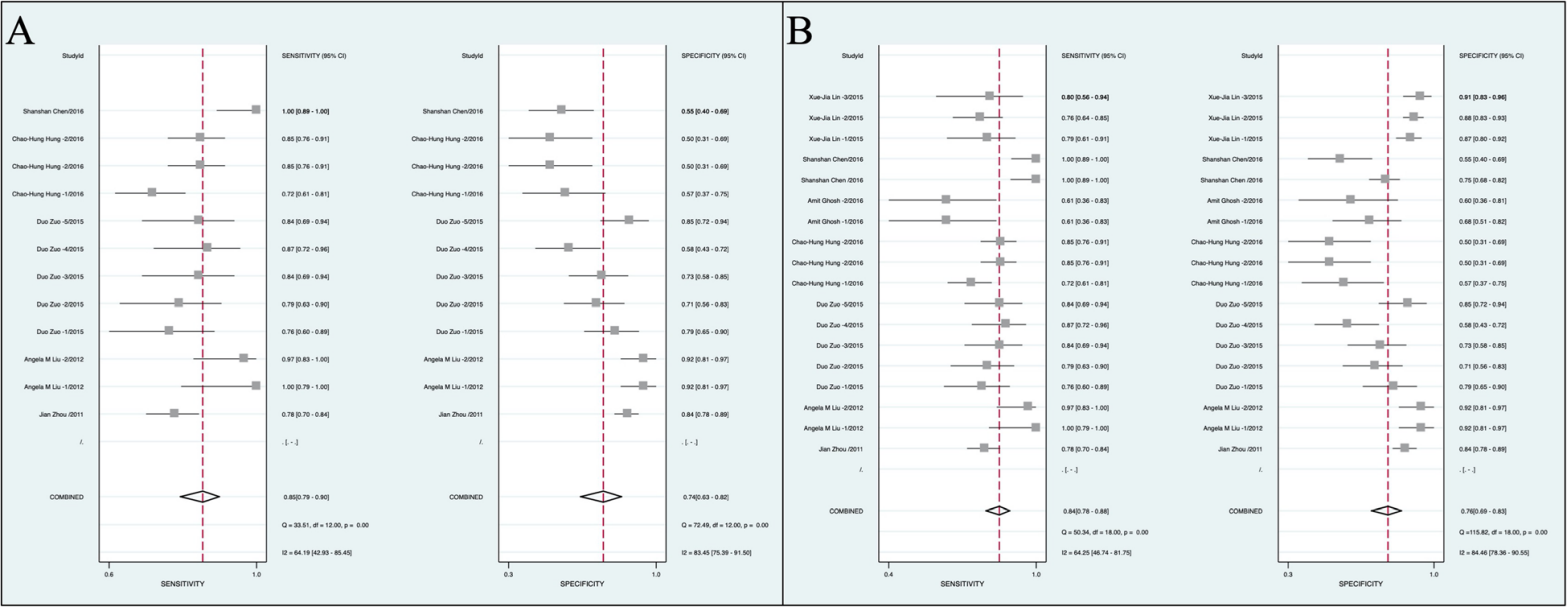
Forest plots show the pooled sensitivity and specificity of circulating miRNAs in discriminating HBV-HCC patients with AFP<20 ng/ml (**a**) or AFP<400 ng/ml (**b**) from non-HCC controls

A total of 18 data sets from 8 articles involving 869 HBV-HCC patients and 1338 non-HCC controls were included in the pooled analysis of discriminating HBV-HCC patients with low AFP level from non-HCC controls. For HBV-HCC patients with AFP levels less than 20 ng/ml, the overall SEN and SPE of circulating miRNAs were 0.85 (95% CI: 0.79–0.90) and 0.74 (95% CI: 0.63–0.82), respectively. The corresponding AUC value was 0.88 (95% CI: 0.85–0.90) in the overall sROC curves. For patients with AFP level less than 400 ng/ml, the overall SEN and SPE of circulating miRNAs were 0.84 (95% CI: 0.78–0.88) and 0.76 (95% CI: 0.69–0.83), respectively. The AUC was 0.88 (95% CI: 0.84–0.90). These results suggested a relatively high diagnostic accuracy of circulating miRNAs. The results are detailed in Fig. 2a-b and Fig. 3. In addition to the above pooled analysis, we also summarized the diagnostic SEN, SPE and AUC of 8 different single miRNAs and 5 miRNAs panels involved in the 8 included studies, the results are detailed in Table 2.

**Table 2:** Diagnostic accuracy of circulating miRNAs in HBV-HCC patients with low AFP level or AFP negative HBV-HCC patients

miRNA	AFP level	number of data sets	number of HCC	number of non-HCC	SEN (95%CI)	SPE (95%CI)	AUC (95%CI)
15b, 130b	<400 ng/ml	1	16	59	1	0.92	0.98
15b, 130b	<20 ng/ml	1	30	59	0.97	0.92	0.98
125b	<20 ng/ml	1	38	48	0.76	0.79	0.78
125b	<400 ng/ml	1	31	158	1	0.76	0.94
223	<20 ng/ml	1	38	48	0.79	0.70	0.76
27a	<20 ng/ml	1	38	48	0.84	0.72	0.83
26a	<20 ng/ml	1	38	48	0.87	0.57	0.73
122	<20 ng/ml	1	92	30	0.71	0.58	0.63
7b	<20 ng/ml	1	92	30	0.85	0.51	0.65
122, 7b	<20 ng/ml	1	92	30	0.85	0.50	0.65
126	<400 ng/ml	2	36	58	0.61	0.66	0.50
205	<400 ng/ml	1	32	49	1	0.56	0.82
125b, 223, 27a, 26a	<20 ng/ml	1	38	48	0.84	0.85	0.87
122, 223, 26a, 27a, 192, 21, 801	<400 ng/ml	1	139	187	0.78	0.85	0.88
29a, 29c, 133a, 143, 145, 192, 505	<20 ng/ml	3	119	438	0.77	0.88	0.64
overall	<20 ng/ml	12	615	827	0.85 (0.79–0.90)	0.74 (0.63–0.82)	0.88 (0.85–0.90)
overall	<400 ng/ml	18	869	1338	0.84 (0.78–0.88)	0.76 (0.69–0.83)	0.88 (0.84–0.90)

*miRNAs* microRNAs, *AFP* alpha-fetoprotein, *HBV* hepatitis B virus, *HCC* hepatocellular carcinoma, *HBV-HCC* hepatitis B virus-associated HCC, *SEN* sensitivity, *SPE* specificity, *AUC* area under the curve

### Results of subgroup analysis and sensitivity analysis

Since heterogeneity was presented in the pooled diagnostic accuracy analysis, we performed additional subgroup analysis to assess the potential source of heterogeneity. We divided the study population into different subgroups according to the type of specimen and conference test, study design, miRNA profiling and QUADAS score, but the value of I^2^ of each subgroup was still greater than 50%, which indicated that the factors mentioned above were not the source of heterogeneity. The pooled SEN, SPE, AUC, and I^2^ value of each group are detailed in Table 3.

**Table 3:** Subgroup analysis of the diagnostic accuracy for circulating miRNA in HBV-HCC patients with low AFP

	SEN (95%CI)	SPE (95%CI)	AUC (95%CI)	number of data sets	number of HCC	number of non-HCC	heterogeneity (OR-I^**2**^)
**Specimen**							
Serum	0.83 (0.78–0.86)	0.79 (0.69–0.86)	0.87 (0.83–0.89)	13	631	886	69.7%
Plasma	0.92 (0.52–0.99)	0.72 (0.61–0.81)	0.80 (0.77–0.84)	5	238	452	79.2%
**Conference test**							
histology	0.85 (0.79–0.90)	0.74 (0.63–0.82)	0.88 (0.85–0.90)	12	683	684	69.0%
histology or imaging	0.79 (0.64–0.89)	0.81 (0.72–0.88)	0.87 (0.84–0.90)	6	186	654	77.2%
**Study design**							
case-control	0.84 (0.84–0.84)	0.67 (0.67–0.67)	0.80 (0.76–0.83)	12	565	595	58.3%
cohort	0.85 (0.74–0.92)	0.89 (0.85–0.91)	0.92 (0.90–0.94)	6	304	743	48.3%
**miRNAs profiling**							
single	0.84 (0.73–0.91)	0.66 (0.60–0.72)	0.76 (0.72–0.79)	10	435	517	56.0%
panel	0.83 (0.76–0.88)	0.86 (0.79–0.91)	0.89 (0.86–0.92)	8	434	821	63.5%
**QUADAS score**							
≥ 9	0.85 (0.77–0.90)	0.81 (0.73–0.88)	0.90 (0.87–0.92)	8	375	545	53.8%
<9	0.83 (0.72–0.90)	0.72 (0.60–0.81)	0.85 (0.81–0.88)	10	494	793	76.2%

*HBV* hepatitis B virus, *HCC* hepatocellular carcinoma, *HBV-HCC*, hepatitis B virus-associated HCC, *AFP* alpha-fetoprotein, *miRNAs* microRNAs, *SEN* sensitivity, *SPE* specificity, *AUC* area under the curve, *OR* odds ratio *QUADAS*. Quality Assessment of Diagnostic Accuracy Studies

The process of sensitivity analysis was to remove each individual study one by one and to check whether the overall outcome of the remaining studies changed significantly. It is the main method for detecting the stability of results. Our sensitivity analysis showed that the overall outcome did not change significantly after removing any of the individual studies, indicating that the results were stable.

### Publication bias

The *p* value of Deeks’ funnel plots was 0.87 (> 0.05), indicating no publication bias exists (Fig. 2c).

## Discussion

Currently, AFP analysis exhibits an unsatisfactory diagnostic performance in HCC patients. The SEN of AFP in the diagnosis of HCC is about 60 to 70%, which means that the diagnosis of 30 to 40% of HCC patients will be missed. On the other hand, AFP levels are often elevated in patients with chronic liver diseases, such as hepatitis and cirrhosis [26]. Therefore, it is of great importance to develop a novel diagnostic marker which could complement AFP.

MiRNAs are involved in various physiological and pathological processes in vivo. Compared with mRNAs, miRNAs are more stable and not easily degraded in body fluids because of their high resistance to RNase activity, as well as to extreme pH and temperature [27, 28], indicating that the aberrant expression of miRNAs seems to be a promising candidate to fill this need for an additional diagnostic tool. However, circulating miRNAs determination is not a widely accessible technique on clinical grounds, there are still challenges to overcome before clinical application: (1) The isolation and purification of samples require high proficiency. Unlike intercellular miRNAs, circulating miRNAs need to be more cautious when centrifuged from peripheral blood sample [29]; (2) Circulating miRNAs can be accurately detected and quantified by RT-PCR, microarray or next-generation sequencing (NGS) [30]. Therefore, it is indispensable to unify the measurement methods and eliminate the deviation; (3) In addition to the technical challenges, the precise function and biology characteristics of circulating miRNAs in HCC still remain more investigations before clinical transformation [31, 32].

The present meta-analysis assessed the diagnostic efficiency of circulating miRNAs in differentiating HBV-HCC patients with low AFP levels from non-HCC controls. The promising finding is that for HBV-HCC patients with AFP levels less than 20 ng/ml, the overall SEN and SPE of circulating miRNAs were 0.85 (95% CI: 0.79–0.90) and 0.74 (95% CI: 0.63–0.82), respectively. The corresponding AUC value was 0.88 (95% CI: 0.85–0.90) in the overall sROC curves. For HBV-HCC patients with AFP levels less than 400 ng/ml, the overall SEN and SPE of circulating miRNAs were 0.84 (95% CI: 0.78–0.88) and 0.76 (95% CI: 0.69–0.83), respectively. The AUC was 0.88 (95% CI: 0.84–0.90). The abscissa of the ROC curve is (1-specificity) while the ordinate is sensitivity, so the closer the curve is to the upper left corner, the greater the SEN and SPE, and the greater the corresponding AUC [33]. When the AUC exceeds 0.8, the diagnostic test is considered to have a satisfactory diagnostic efficiency; if the AUC exceeds 0.9, the diagnostic accuracy is very high. Therefore, circulating miRNAs were shown to have good diagnostic power. In the present meta-analysis, a total of 8 different single miRNAs and 5 miRNAs panels were mentioned in our included studies, we summarized their diagnostic accuracy. For HBV-HCC patients with AFP levels less than 400 ng/ml, miR-125b and miR-205 exhibited a high SEN of more than 90% while the combination of miR-15b and miR-130b showed high diagnostic accuracy with both SEN and SPE exceeding 90%. For those with AFP levels less than 20 ng/ml, miR-26a, 27a, 7b as well as the combination of miR-122 and miR-7b exhibited a SEN of more than 80% while the combination of miR-29a, 29c, 133a, 143, 145, 192 and 505 yielded a SPE of more than 80%. In addition, the combination of miR-15b and miR-130b showed high diagnostic accuracy with both SEN and SPE exceeding 90%. These study results suggest that circulating miRNAs may be an ideal novel diagnostic biomarker for HBV-HCC patients with low AFP levels, because circulating miRNAs are able to discriminate cases of HBV-HCC that cannot be detected by the conventional AFP testing. The measurement of circulating miRNAs as a second-line test could be a remedy for the diagnosis of low-AFP HBV-HCC.

Heterogeneity, also known as dissimilarity, is defined as the differences between the studies included in a meta-analysis. The findings of our meta-analysis also confirmed the existence of heterogeneity. Threshold effect and non-threshold effect are two main sources of heterogeneity. We first performed Spearman correlation analysis to verify the existence of a threshold effect. The results showed that the Spearman correlation coefficient was 0.005 and the corresponding *p* value was 0.984 (> 0.05); therefore, the existence of a threshold effect was excluded. Next, additional subgroup analysis on the basis of the type of specimen and conference test, study design, the usage of miRNAs, and QUADAS score were performed to assess other potential sources of heterogeneity. The results indicated that the factors mentioned above were not the source of heterogeneity, suggesting that the influencing factors are complex. In the sensitivity analysis, the results suggested that the results were stable. There was also no evidence of publication bias in the present meta-analysis. Therefore, we speculated that it was the variance in the type of miRNAs and normalization controls involved in the included studies that contributed to bias.

There were some limitations in the present meta-analysis. First of all, a high-quality diagnostic study should avoid case-control design and inappropriate exclusions. In other words, a diagnostic study with high quality should include not only patients with a confirmed diagnosis, but also some patients with suspected disease (difficult-to-diagnose patients); otherwise, the efficiency of diagnostic tests may be exaggerated to some extent [14]. Secondly, heterogeneity is a common situation in meta-analysis of diagnostic tests [34, 35]; it also presented in our meta-analysis. We failed to identify sources of heterogeneity even though sensitivity analysis and subgroup analysis were performed.

The present study also had some strengths. First, we formulated a scientific and complete search strategy without restricting language and publication year. Eight eligible studies were ultimately included. The second strength of the present study is that it evaluated the diagnostic performance of circulating miRNAs in HBV-HCC patients with low AFP levels, which had not previously been investigated. Third, not only have we determined the pooled SEN, SPE and AUC of circulating miRNAs in differentiating HBV-HCC patients with low AFP levels from non-HCC controls by pooled analysis, but we have also summarized the diagnostic accuracy of different miRNAs involved in all the included studies in detail, providing valuable information for further scientific research and clinical application.

## Conclusions

In conclusion, the results of the present systematic review and meta-analysis indicate that the use of circulating miRNAs has satisfactory diagnostic accuracy for HBV-HCC patients with low AFP levels and provides a biomarker comparable to AFP. The use of circulating miRNAs holds potential value as a novel biomarker for the diagnosis of low-AFP HBV-HCC.

## Data Availability

Please contact author for data requests.

## Abbreviations

HCC: Hepatocellular carcinoma
HBV-HCC: Hepatitis B virus-associated HCC
US: Ultrasound
AFP: Alpha-fetoprotein
CNKI: Chinese National Knowledge Infrastructure
SEN: Sensitivity
SPE: Specificity
TP: True positive
TN: True negative
FP: False positive
FN: False negative
PCR: Polymerase chain reaction
CI: Confidence interval
AUC: Area under the curve
RT-PCR: Real-time PCR
NGS: Next-generation sequencing
